# Supplementary material for: Exploiting a Shortcoming of Coupled-Cluster Theory: The Extent of Non-Hermiticity as a Diagnostic Indicator of Computational Accuracy
Source: J Phys Chem Lett. 2025 May 14;16(20):5121–7. doi: 10.1021/acs.jpclett.5c00885 (PMC12108035; doi:10.1021/acs.jpclett.5c00885)
Supplement: Supplementary file 1 [file jz5c00885_si_001.pdf]

SUPPORTING INFORMATION

Exploiting a Shortcoming of Coupled-Cluster Theory: The Extent of non-Hermiticity as a Diagnostic Indicator of Computational Accuracy. [jz-2025-00885n.R1]

Kaila E. Weflen,[†] Megan R. Bentley,[†] James H. Thorpe,[‡] Peter R. Franke,[†] Jan M. L. Martin,[*,†,¶] Devin A. Matthews,[‡] and John F. Stanton[†,§]

†*Quantum Theory Project, Department of Chemistry, University of Florida, Gainesville, FL 32611*

‡*Department of Chemistry, Southern Methodist University, Dallas, TX 75275*

¶*On sabbatical from: Department of Molecular Chemistry and Materials Science, Weizmann Institute of Science, 7610001 Reḥovot, Israel.*

§*Senior author: Deceased March 21, 2025*

E-mail: gershom@weizmann.ac.il

Phone: +972 8 9342533 or +1 352 7217134. Fax: +972 8 9343029

Supporting Information

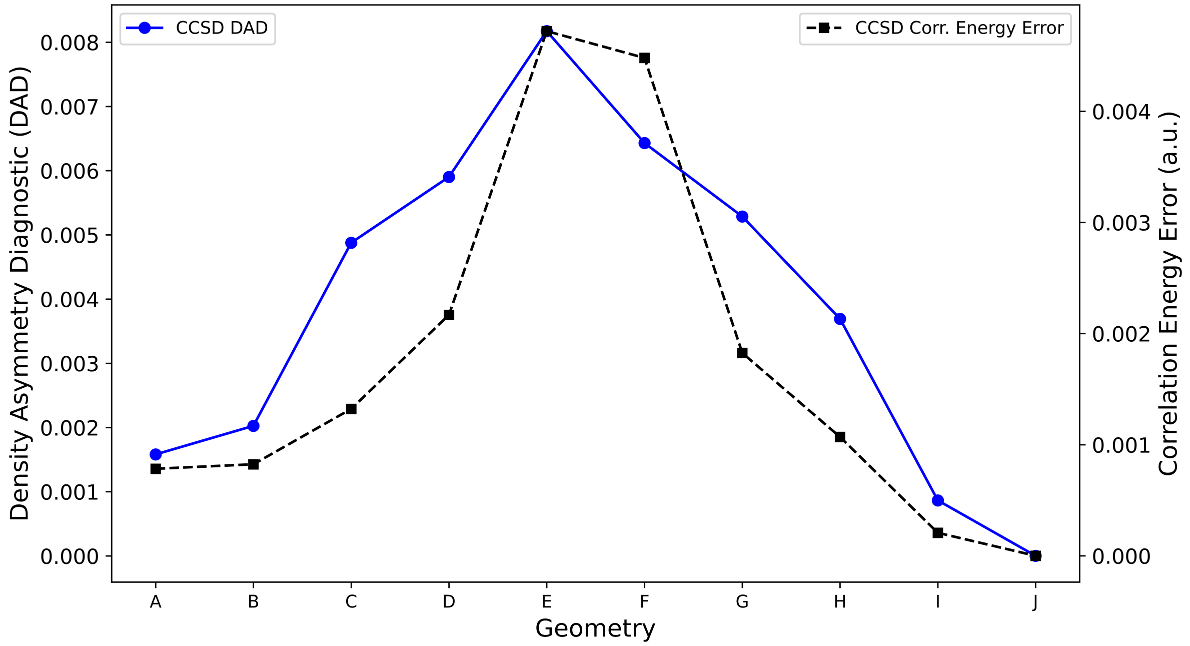

Figure S1: Density Asymmetry Diagnostic and Correlation Energy Error (as compared to CCSDTQ) for the insertion of Be into H_2 at points A-J given in Ref. 1 at the frozen-core CCSD/cc-pVTZ approximation.

Table S1: Associated DAD and Correlation Energy values for the insertion of Be into H_2 in the frozen-core approximation at CCSD, CCSDT, and CCSDTQ levels of theory using cc-pVTZ basis set. The DAD value evaluated at CCSDTQ is zero for every point as CCSDTQ provides the exact treatment.

Point	DAD CCSD	DAD CCSDT	E(CCSD)	E(CCSDT)	E(CCSDTQ)
A	0.0015815	0.0000934	-15.84748	-15.84825	-15.84827
B	0.0020260	0.0001389	-15.81684	-15.81766	-15.81767
C	0.0048773	0.0005262	-15.75078	-15.75208	-15.75210
D	0.0059024	0.0008820	-15.69554	-15.69767	-15.69770
E	0.0081730	0.0021719	-15.66616	-15.67107	-15.67088
F	0.0064304	0.0020927	-15.67219	-15.67645	-15.67667
G	0.0052863	0.0003863	-15.73276	-15.73451	-15.73458
H	0.0036927	0.0002129	-15.77101	-15.77204	-15.77208
I	0.0008653	0.0000446	-15.78930	-15.78950	-15.78951
J	0.0000021	0.0000003	-15.79076	-15.79076	-15.79076

Table S2: Comparison of DAD and Energy Errors for the molecules in Table II. The cc-pVDZ basis set was used throughout. Unlike in Table II, (1s) core orbitals were frozen.

Mol-	DAD diagnostic			Error from full CI			E(FCI)
ecule	CCSD	CCSDT	CCSDTQ	CCSD	CCSDT	CCSDTQ	
BH_4^-	0.0038991	0.0002657	0.0000184	0.003624	0.000096	0.000004	-27.138 787
CH_4	0.0029586	0.0001753	0.0000197	0.004231	0.000158	0.000010	-40.387 346
NH_3	0.0022457	0.0001472	0.0000226	0.004409	0.000337	0.000020	-56.402 658
H_2O	0.0021959	0.0002036	0.0000195	0.003680	0.000473	0.000018	-76.241 750
HF	0.0032242	0.0001948	0.0000369	0.002412	0.000404	0.000011	-100.228 634
BN	0.0562656	0.0242503	0.0053528	0.028542	0.002455	0.000270	-79.205 042
C_2	0.0143525	0.0018348	0.0004776	0.029475	0.003229	0.000619	-75.728 803
N_2	0.0053171	0.0005790	0.0001290	0.013696	0.001674	0.000201	-109.277 316
CO	0.0148615	0.0030954	0.0004130	0.012242	0.001019	0.000060	-113.056 132
BF	0.0110149	0.0017292	0.0001465	0.006878	0.000523	0.000017	-124.380 264
O_3	0.0094080	0.0026562	0.0004983	0.039298	0.006184	0.000785	-224.915 693

A full CI calculation on ozone was infeasible: the FCI energy given above was obtained by additivity approximation as detailed in the text.

Table S3: Coefficients of determination R^2 between different diagnostics for the closed-shell subset of the W4-11 thermochemical benchmark dataset

	DAD	T_1^{diag}	D_1^{diag}	Max(t_{ia})	T_4^{diag}	T_3^{diag}	S_{norm}	$I_{\overline{\mathrm{ND}}}$	(A-1)/N	$\mathrm{T}_2^{\mathrm{diag}}$	$\mathrm{M}^{\mathrm{diag}}$	$\mathrm{D}_2^{\mathrm{diag}}$	$I_{\mathrm{ND}}^{\max}$	Max(t_{ijab})	%TAE$_{\mathrm{corr}}$	%TAE[(T)]	%TAE[(Q)]	%TAE[ΔX,SC-HF]	%TAE[ΔX]
DAD	1.00	0.86	0.76	0.74	0.77	0.72	0.08	0.14	0.31	0.35	0.24	0.20	0.19	0.19	0.07	0.22	0.23	0.03	0.06
T_1^{diag}	0.86	1.00	0.89	0.87	0.86	0.85	0.19	0.30	0.51	0.57	0.50	0.44	0.44	0.38	0.21	0.42	0.45	0.12	0.17
D_1^{diag}	0.76	0.89	1.00	0.96	0.74	0.74	0.07	0.15	0.31	0.38	0.43	0.35	0.34	0.34	0.24	0.45	0.47	0.15	0.20
Max(t_{ia})	0.74	0.87	0.96	1.00	0.74	0.73	0.08	0.16	0.32	0.37	0.42	0.34	0.33	0.35	0.20	0.41	0.44	0.12	0.16
T_4^{diag}	0.77	0.86	0.74	0.74	1.00	0.94	0.20	0.31	0.47	0.54	0.51	0.42	0.43	0.39	0.19	0.42	0.46	0.15	0.16
T_3^{diag}	0.72	0.85	0.74	0.73	0.94	1.00	0.23	0.34	0.49	0.61	0.57	0.47	0.49	0.41	0.18	0.40	0.46	0.16	0.15
S_{norm}	0.08	0.19	0.07	0.08	0.20	0.23	1.00	0.96	0.84	0.61	0.39	0.50	0.51	0.26	0.00	0.04	0.08	0.00	0.00
$I_{\overline{\mathrm{ND}}}$	0.14	0.30	0.15	0.16	0.31	0.34	0.96	1.00	0.94	0.75	0.55	0.62	0.65	0.38	0.03	0.10	0.16	0.01	0.01
$(A-1)/N$	0.31	0.51	0.31	0.32	0.47	0.49	0.84	0.94	1.00	0.85	0.64	0.67	0.70	0.45	0.07	0.19	0.26	0.03	0.03
T_2^{diag}	0.35	0.57	0.38	0.37	0.54	0.61	0.61	0.75	0.85	1.00	0.73	0.72	0.75	0.57	0.09	0.21	0.28	0.08	0.07
M^{diag}	0.24	0.50	0.43	0.42	0.51	0.57	0.39	0.55	0.64	0.73	1.00	0.90	0.94	0.81	0.33	0.49	0.56	0.28	0.28
D_2^{diag}	0.20	0.44	0.35	0.34	0.42	0.47	0.50	0.62	0.67	0.72	0.90	1.00	0.98	0.78	0.26	0.38	0.44	0.19	0.21
$I_{\mathrm{ND}}^{\max}$	0.19	0.44	0.34	0.33	0.43	0.49	0.51	0.65	0.70	0.75	0.94	0.98	1.00	0.77	0.25	0.38	0.46	0.19	0.20
Max(t_{ijab})	0.19	0.38	0.34	0.35	0.39	0.41	0.26	0.38	0.45	0.57	0.81	0.78	0.77	1.00	0.28	0.38	0.44	0.27	0.25
%TAE$_{\mathrm{corr}}$	0.07	0.21	0.24	0.20	0.19	0.18	0.00	0.03	0.07	0.09	0.33	0.26	0.25	0.28	1.00	0.90	0.80	0.89	0.97
%TAE[(T)]	0.22	0.42	0.45	0.41	0.42	0.40	0.04	0.10	0.19	0.21	0.49	0.38	0.38	0.38	0.90	1.00	0.96	0.75	0.83
%TAE[(Q)]	0.23	0.45	0.47	0.44	0.46	0.46	0.08	0.16	0.26	0.28	0.56	0.44	0.46	0.44	0.80	0.96	1.00	0.66	0.73
%TAE[ΔX,SC-HF]	0.03	0.12	0.15	0.12	0.15	0.16	0.00	0.01	0.03	0.08	0.28	0.19	0.19	0.27	0.89	0.75	0.66	1.00	0.91
%TAE[ΔX]	0.06	0.17	0.20	0.16	0.16	0.15	0.00	0.01	0.03	0.07	0.28	0.21	0.20	0.25	0.97	0.83	0.73	0.91	1.00

The diagnostics were evaluated for the closed-shell subset of the W4-11 thermochemical benchmark dataset.[10]

- $T_1^{\rm diag} = \sqrt{\vec{t_1}^T \vec{t_1}/N_{\rm corr}}$;[2] $T_2^{\rm diag}$, $T_3^{\rm diag}$, $T_4^{\rm diag}$ defined by analogy for the doubles, triples, and quadruples amplitudes, respectively, all obtained from CCSDTQ/cc-pVDZ calculations.

- $D_1^{\rm diag} = \sqrt{\lambda_{\rm max}(\vec{t_1}.\vec{t_1}^T)}$ as defined by Nielsen and Janssen[3] and obtained at the CCSD/cc-pVTZ level using MOLPRO.[5]

- $D_2^{\rm diag} = \sqrt{\lambda_{\rm max}(\vec{t_2}.\vec{t_2}^T)}$ as defined by Nielsen and Janssen[4] and obtained at the CCSD/cc-pVTZ level using MOLPRO.[5]

- $M_{\rm diag} = (2 - n_{\rm HOMO} + n_{\rm LUMO})/2$ is the Truhlar M diagnostic, where n_i are the natural orbital occupations from (in this case) a CCSD/cc-pVTZ calculation.

- $I_{\overline{\rm ND}}$ and $I_{\rm ND}^{\rm max}$ as defined by Matito and coworkers.[7]

- Max(t_{ia}) and Max(t_{ijab}) are the largest (in absolute value) singles and doubles amplitudes, respectively.

- %TAE$_{\rm corr}$ is the percentage of the molecular total atomization energy (TAE) accounted for by electron correlation, i.e., 100%-%TAE(SCF), as introduced in Ref. 8. Obtained at the CCSD(T)/cc-pVTZ level.

- %TAE[(T)] is the percentage of the above TAE accounted for by parenthetical triples, likewise introduced as a diagnostic in Ref. 8.

- %TAE[(Q)] is the percentual contribution of connected quadruples to the atomization energy, as a proxy for post-CCSD(T) correlation. Likewise introduced in Ref. 8.

- %TAE[ΔX] is the percentage of a TAE obtained at the DFT level (in this case, TPSS) that stems from the exchange functional. Introduced in Ref. 9

- %TAE[ΔX,SC-HF] instead, take the percentual difference between the exchange functional contribution to TAE evaluated with self-consistent Kohn-Sham orbitals, and the same evaluated for Hartree-Fock orbitals. This variant[9] avoids the somewhat 'heretical' concept of an atomization energy corresponding to a Hartree product (which is not antisymmetrized). For cases with predominantly dynamical correlation, the HF and KS densities are very similar, while they are quite different for cases with strong static correlation.[12]

- S_{norm} is the von Neumann-like correlation entropy[11] normalized by the number of correlated orbitals.

Table S4: The T_1^{diag} diagnostics and its analogues for double, triple, and quadruple substitutions for the molecules in Table II, as well as the largest n-tuple excitation amplitudes for n=1–4. The underlying electronic structure calculations were carried out at the CCSDTQ/cc-pVDZ level with (1s) cores frozen

Molecule	T_1^{max}	T_2^{max}	T_3^{max}	T_4^{max}	T_1^{diag}	T_2^{diag}	T_3^{diag}	T_4^{diag}
BH_4^-	0.0092(×6)	0.0401(×2)	0.0017(×4)	0.0001(×4)	0.0074	0.0740	0.0089	0.0010
CH_4	0.0046(×6)	0.0332(×2)	0.0014(×4)	0.0001(×2)	0.0047	0.0696	0.0081	0.0008
NH_3	0.0063(×4)	0.0484	0.0014(×4)	0.0002	0.0053	0.0665	0.0074	0.0007
H_2O	0.0074(×2)	0.0519	0.0014(×2)	0.0002	0.0057	0.0607	0.0062	0.0009
HF	0.0080(×2)	0.0485	0.0027(×4)	0.0003(×4)	0.0051	0.0523	0.0045	0.0007
BN	0.2151(×2)	0.3458	0.0463(×4)	0.0121(×2)	0.0817	0.1426	0.0386	0.0122
C_2	0.0850(×2)	0.3988	0.0193(×4)	0.0060(×2)	0.0374	0.1537	0.0298	0.0080
N_2	0.0183(×5)	0.1134(×2)	0.0058(×4)	0.0038	0.0109	0.0798	0.0125	0.0025
CO	0.0304(×5)	0.0862(×2)	0.0133(×5)	0.0043	0.0174	0.0768	0.0138	0.0031
BF	0.0300(×4)	0.1192(×2)	0.0056(×4)	0.0018	0.0164	0.0745	0.0104	0.0024
O_3	0.0859(×2)	0.3140	0.0231(×2)	0.0020(×2)	0.0298	0.0889	0.0203	0.0048

Suffixes (×n) indicate there are n symmetry-equivalent excitations with the same amplitude value. Absence means $n = 1$.

References

(1) Purvis III, G.D.; Shepard, R.; Brown, F.B.; Bartlett, R.J., C_{2v} Insertion pathway for BeH_2: A test problem for the coupled-cluster single and double excitation model, *Int. J. Quantum Chem.* **1983**, *23*, 835-845.

(2) Lee, T. J.; Taylor, P. R. A Diagnostic for Determining the Quality of Single-Reference Electron Correlation Methods. *Int. J. Quantum Chem. Symp.* **1989**, *23*, 199–207.

(3) Janssen, C. L.; Nielsen, I. M. B. New Diagnostics for Coupled-Cluster and Møller–Plesset Perturbation Theory, *Chem. Phys. Lett.* **1998**, *290*, 423–430.

(4) Nielsen, I. M. B.; Janssen, C. L. Double-substitution-based Diagnostics for Coupled-Cluster and Møller–Plesset Perturbation Theory, *Chem. Phys. Lett.* **1999**, *310*, 568–576.

(5) Werner, H.-J.; Knowles, P. J.; Manby, F. R.; Black, J. A.; Doll, K.; Heßelmann, A.; Kats, D.; Köhn, A.; Korona, T.; Kreplin, D. A.; Ma, Q.; Miller, T. F.; Mitrushchenkov, A.; Peterson, K. A.; Polyak, I.; Rauhut, G.; Sibaev, M., The Molpro quantum chemistry package, *J. Chem. Phys.* **2020**, *152*, 144107.

(6) Tishchenko, O.; Zheng, J.; Truhlar, D. G. Multireference model chemistries for thermochemical kinetics. *J. Chem. Theory Comput.* **2008**, *4*, 1208–1219 (2008).

(7) Xu, X.; Soriano-Agueda, L.; López, X.; Ramos-Cordoba, E.; Matito, E. An All-Purpose Measure of Electron Correlation for Multireference Diagnostics. *J. Chem. Theory Comput.* **2024**, *20*, 721–727.

(8) Karton, A.; Rabinovich, E.; Martin, J. M. L.; Ruscic, B. W4 theory for computational thermochemistry: In pursuit of confident sub-kJ/mol predictions. *J. Chem. Phys.* **2006**, *125*, 144108.

(9) Martin, J. M. L.; Santra, G.; Semidalas, E. An exchange-based diagnostic for static correlation, *AIP Conference Proceedings* **2022**, *2611*, 020014, available also at http://arxiv.org/abs/2111.01879

(10) Karton, A.; Daon, S.; Martin, Jan M.L. W4-11: a high-confidence benchmark dataset for computational thermochemistry derived from first-principles W4 data, *Chem. Phys. Lett.* **2011**, *510*, 165–178.

(11) Ziesche, P. Correlation Strength and Information Entropy *Int. J. Quantum Chem.* **1995** *56*, 363-369.

(12) Handy, N. C.; Cohen, A. J. Left-right correlation energy, *Mol. Phys.* **2001**, *99*, 403-412.
